# A Randomized Comparative Trial of the Knowledge Retention and Usage Conditions in Undergraduate Medical Students Using Podcasts and Blog Posts

**DOI:** 10.7759/cureus.2065

**Published:** 2018-01-15

**Authors:** Kelly Lien, Alvin Chin, Anton Helman, Teresa M Chan

**Affiliations:** 1 Michael G. Degroote School of Medicine, McMaster University; 2 Faculty of Health Sciences, Department of Medicine, Division of Emergency Medicine, McMaster University; 3 Department of Family and Community Medicine, University of Toronto

**Keywords:** podcasts, blog posts, medical education, asynchronous education, free medical online education, online learning resources, undergraduate medical education

## Abstract

Introduction

Podcasts and blog posts have gained popularity in Free Open Access Medical education (FOAM). Previous work suggests that podcasts may be useful for knowledge acquisition in undergraduate medical education. However, there remains a paucity of research comparing the two mediums. This study aims to investigate if there are differences in knowledge acquisition and usage conditions by medical students using podcasts and blog posts.

Methods

Medical students were randomized to either the podcast or blog post group. They completed an initial online assessment of their baseline knowledge on the subject matter. Participants then received access to learning materials and were given four weeks to complete the follow-up assessment on their own time. Independent t-test, paired samples t-test, and a mixed ANOVA (analysis of variance) were conducted to assess knowledge acquisition. An intention-to-teach analysis was used to impute missing data from students lost to follow-up. Simple descriptive statistical data was used to describe media usage conditions.

Results

Completion of at least one follow-up assessment was comparable (68% podcasts (n = 21/31), 73% blog posts (n = 22/30)). Both groups showed significant improvements in their test scores, with an average 22% improvement for the podcast group and 29% for the blog post group. There was no significant statistical difference in knowledge acquisition between educational modalities overall. Students in the blog post group that completed both post-intervention quizzes showed a larger improvement than the podcast group in the toxicology topic, with similar improvements in the asthma topic. The podcast group tended to engage in multiple activities while using the learning materials (e.g. at least two to three of the following: driving, eating, chores, taking notes, exercising/walking), while the blog readers tended to do fewer activities (e.g. only one of the following: note taking, eating).

Conclusion

This study suggests that podcasts and blog posts are useful for extracurricular knowledge acquisition by undergraduate medical students with no significant difference between the two modalities. The usage conditions for each type of media differ.

## Introduction

A core tenet of undergraduate medical education is teaching medical students to be self-directed, lifelong learners [1]. Although medical schools often recommend a reading list of textbooks and primary literature, free open access medical education (FOAM) [2] has rapidly gained popularity with medical trainees, as this cohort increasingly relies on the internet to obtain up to date medical information [3-5]. FOAM encompasses a continually expanding database of resources for medical education, such as podcasts, blog posts, videos, and Twitter feeds.

Podcasts and blog posts are both increasingly being used in medical education [6-9]. Both are inexpensive to produce, easy to distribute, and offer great portability compared to traditional lectures. In addition, there is some evidence that these forms of media offer better learner engagement compared to traditional didactic lectures [5, 10-12]. Several residency programs, such as emergency medicine, are now incorporating newer teaching methods that include short interactive lectures, small group sessions and asynchronous components, such as blog posts and podcasts [13-15].

Although these resources are being incorporated by medical learners into their education, there is a paucity of research so far demonstrating their efficacy in learning outcomes. Many studies conducted in the literature so far lack a record of the knowledge gained as a measure of objective learning benefit [16-18] and, if it is recorded, combine podcasts and/or blog posts with further methods of teaching [11, 19-20]. Furthermore, these studies are often conducted with dental or nursing students rather than undergraduate medical students. A PubMed search of “medical education” AND “podcasts” found only two controlled trials that measured the effectiveness of podcasts as a tool for knowledge acquisition [10, 19]. Of note, both studies utilized a video podcast style uncommon with most FOAM resources. Schreiber and colleagues found no difference between live lectures versus video podcasts in terms of knowledge acquisition [19], while podcast users scored significantly higher in a post-test evaluation compared to text-based users in the study by Back and colleagues [10].

A recent prospective trial attempted to gain data on the prior utilization, usage conditions, and preferences of medical students regarding podcasts in undergraduate medical education [21]. The study found that students were listening to podcasts while engaging in other activities, such as driving, completing chores, and exercising. The secondary objective of determining knowledge acquisition and retention by students found that participants did experience a significant gain in knowledge overall.

This current study aimed to determine if there were differences in the knowledge acquisition by undergraduate medical students using either podcasts and blog posts. The secondary objective in this study was to obtain data on the extracurricular usage conditions of both types of media, including where and when students used the media, if they were participating in other activities simultaneously, how long they used it for, etc.

## Materials and methods

Participants

Participants consisted of medical students from a Canadian medical school who did not participate in the study by Chin and colleagues [21]. They were recruited through the school’s student mailing lists and the school’s affiliated Facebook group. Students were informed that participation in this study would have no impact on their academic standing, and they submitted their informed consent for inclusion through an online consent form. Approval was gained from the Hamilton Integrated Research Ethics Board and the local protocol review committee of the university.

Study design

We used a randomized trial to compare two groups of learners. The first group was given information in podcast form, and the second group was provided the same information in blog post format. Participants were block-randomized by year of study and randomly assigned to either the podcast or blog post group. Both groups completed two sets of questionnaires: an initial questionnaire and pre-intervention multiple choice test, and a post-test and exit questionnaire. The pre-test questionnaire included questions on the student’s familiarity with podcasts and blog posts, their current usage of the media, and their preference between the two, if applicable. The exit questionnaire included questions regarding the student’s experience with the media type, whether they would consider using the media in the future, and what their preferences were in terms of time or word limits. Both groups were given four weeks to utilize the learning materials and to complete the final evaluation on their own time, which allowed for a measurement of extracurricular media usage patterns. The students were told to complete the exit questionnaire regardless of whether they used the learning materials to aid in the follow-up of participants, as well as to determine if there were any common reasons for not using the media.

The podcast group was provided with audio MP3 files on asthma (57:44 min) and toxicology (36:38 min), produced by AC, AH, and TC from a previous study at our centre [21]. The students were not aware of the learning topics when signing up for the study, only that the topics would be commonly encountered problems in medicine. The blog post group was provided with two website links to blog posts on the same topics, written by a medical student investigator (KL) and placed through the CanadiEM coached peer review process [22]. Blog posts were created based on the podcast content. The first post was entitled "Asthma in the ED" (emergency department) and contained 1,624 words and the second was entitled "A Practical Approach to Toxicology", which contained 1,196 words. The blog posts were written in English, and the podcasts were also recorded in English.

To determine knowledge acquisition, 11 multiple-choice questions were developed for the asthma topic, and 10 were developed for the toxicology topic. Quiz contents were directly extracted from the provided learning material. Two emergency staff physicians reviewed the questions and answers to determine content validity. The same questions were used for the pre- and post-intervention evaluations. The exit questionnaire included questions regarding the activities performed while using the media. Multiple-choice quizzes and questionnaires were offered using Google Forms (Google Inc., Mountain View, CA, USA).

Analysis

Paired-samples t-tests were performed to measure knowledge changes between pre- and post-intervention for each of the two media types. An independent-samples t-test was used to determine if there was any significant difference between initial pre-intervention scores. We used a mixed analysis of variance (M-ANOVA) to compare the effect of media type on learning benefit, with the within-subjects factor as time, and the between-subjects factor as media modality. The topic (asthma, toxicology) was nested under modality. For students who were lost to follow-up, an “intention-to-teach” analysis was performed wherein an individual’s original baseline knowledge score was used as their final quiz score. This was the most conservative method to account for missing data, with an assumption that students would not lose knowledge by using the learning materials and that knowledge would not decay over the length of the study. A separate analysis was conducted for the students that completed both post-intervention tests.

Sample size calculation

This trial was designed as a superiority trial with podcasts as the control arm to be compared to blog posts. Power calculations were performed a priori to determine the required sample size to detect a significant difference in knowledge acquisition between the two groups. As is conventional in the literature, an α of 0.05 and power of 80% were chosen. The mean standard deviation of 15%, as reported by Chin and colleagues, was used as students from that study could be expected to be drawn from the same population as the current one [21]. An increase of 10% in knowledge acquisition was chosen for this study, based on the study by Back and colleagues, which noted an 8.8% difference between podcast and text users [10]. Using these numbers, a sample size of 58 participants was needed.

## Results

**Figure 1:**
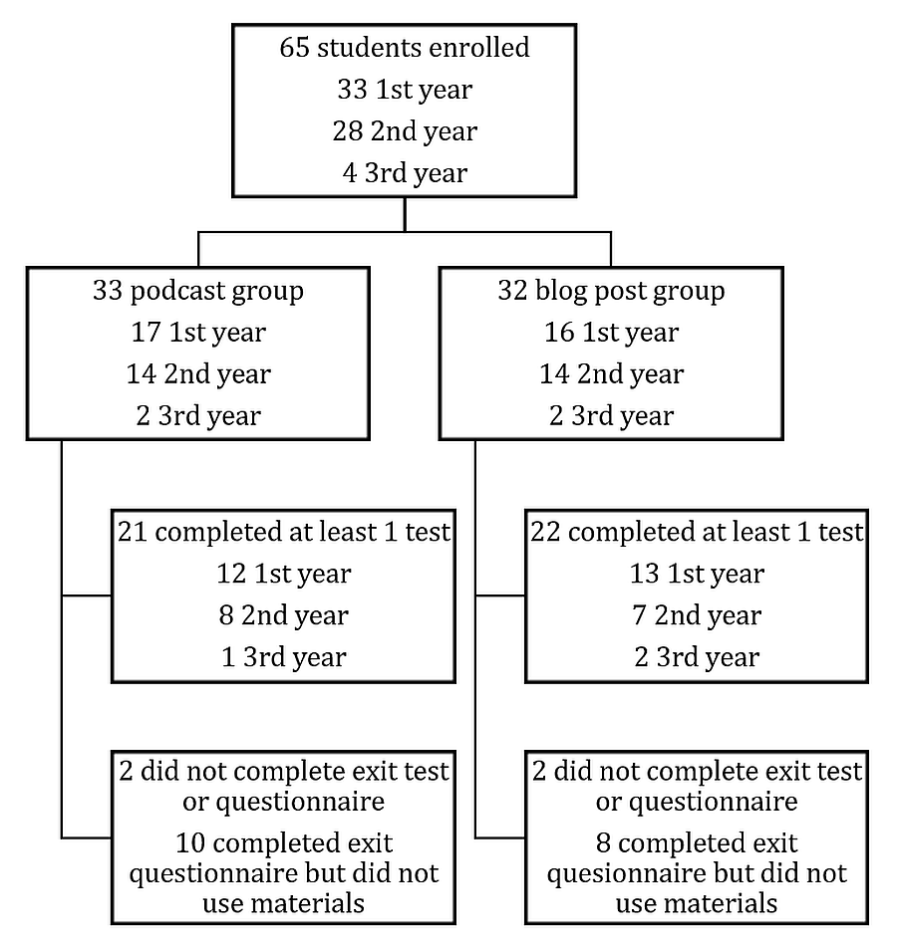
Study enrollment diagram

Our study was conducted from October to December of 2016. Sixty-five medical students enrolled in the study and completed the pre-intervention quiz. First and second-year students were equally represented (33 and 28 students, respectively). Four third-year students participated. Thirty-three students were randomised to the podcast group and 32 to the blog post group (Figure 1).

Second and third-year students were randomized to either group in equal numbers. Two students in each of the two arms did not complete the exit questionnaire and were lost to follow-up. Ten students (32%, n = 31) in the podcast and eight (25%, n = 32) in the blog post group completed the initial pre-intervention quiz but did not utilise the learning materials. These students completed the exit questionnaire stating that they had not used the media and were analysed using the “intention-to-teach” method as described. The scores from these students were imputed into the final calculations as described in the Methods section. Completion of at least one follow-up test was comparable (68% podcasts, 73% blog posts).

Learning outcomes

**Figure 2:**
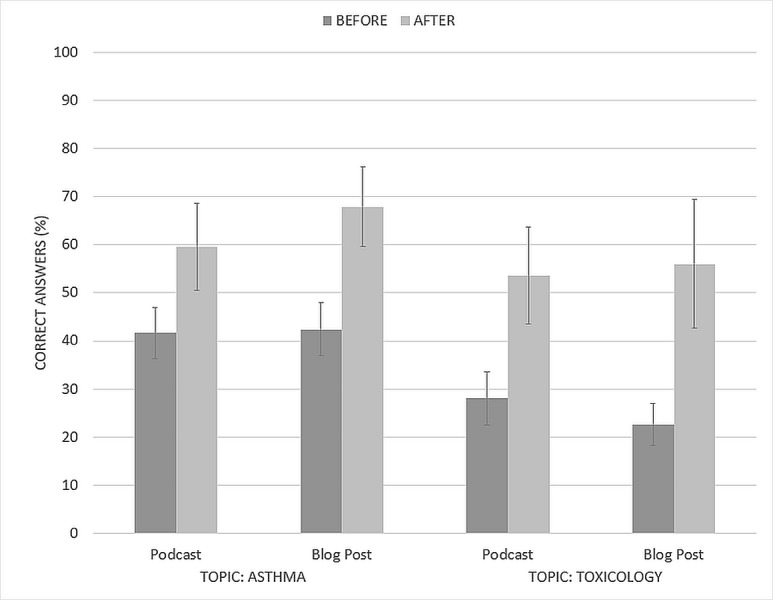
Quiz results for asthma and toxicology topics using intention-to-teach analysis Mean multiple choice quiz results on asthma and toxicology topics before and after usage of podcasts or blog posts for learning by undergraduate medical students (n = 31 for podcasts, n = 30 for blog posts). Error bars represent the 95% confidence interval.

Pre- and post-intervention scores using the intention-to-teach analysis are shown in Figure 2.

The mean pre-intervention asthma quiz score for both groups was 42% (95% confidence interval (CI): ± 5.3% for podcasts, ± 5.6% for blog posts). For the toxicology topic, the pre-intervention quiz scores were 28% and 23% (95% CI: ± 5.5% and ± 4.4%) for the podcast and blog post groups, respectively. There was no significant difference in the pre-intervention scores between the groups (independent-samples t-test, Asthma: p = 0.84; Toxicology: p = 0.13). Both groups of students showed significant improvements in their test scores after using the learning materials. The podcast group improved by 18% and 26% for the Asthma and Toxicology topics, respectively (paired-samples t-test, p < 0.01 for both). Likewise, the blog post group improved by 25% and 33% (paired-samples t-test, p < 0.01 for both). However, there was no significant difference between the two modalities and no discernable interaction between the Topic and the Modality (M-ANOVA, Topic*Modality F(1,59) = 0.001, p = 0.973).  

**Figure 3:**
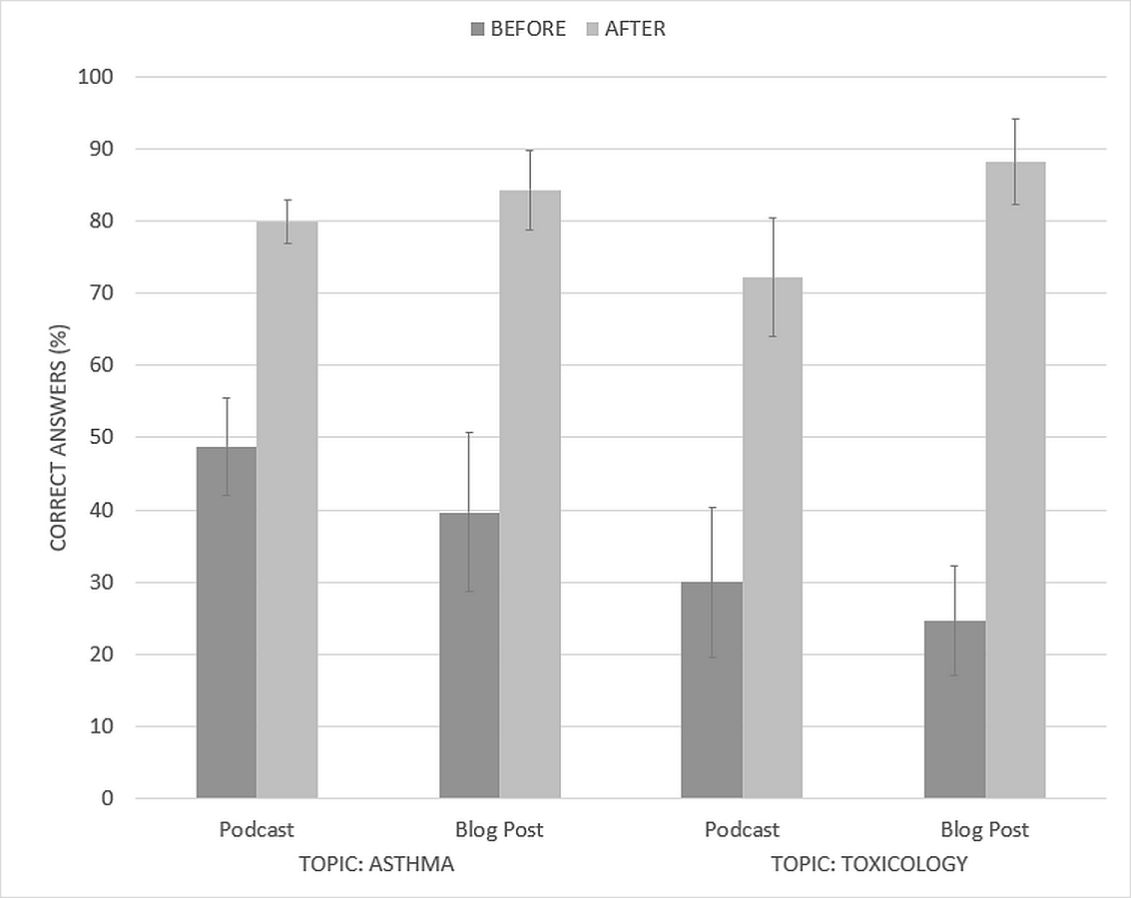
Quiz results for students who completed both asthma and toxicology topics Mean multiple choice quiz results on asthma and toxicology topics before and after usage of podcasts or blog posts for learning by undergraduate medical students who completed both topics (n=14 for podcasts, n=11 for blog posts). Error bars represent the 95% confidence interval.

The pre- and post-intervention scores for students who completed the post-intervention quizzes for both topics are shown in Figure 3 (n = 14 and 11 for the podcast and blog post groups, respectively). 

There was no significant difference in the pre-intervention scores between the groups (Asthma: p = 0.14; Toxicology: p = 0.36). Both groups of students showed significant improvements in their test scores after using the learning materials. The podcast group improved by 31% and 42% for the asthma and toxicology topics, respectively (paired-samples t-test, p < 0.01 for both). Likewise, the blog post group improved by 45% and 64%, respectively (paired-samples t-test, p < 0.01 for both). While the improvements in the asthma topic were similar, there was a statistically significantly greater improvement for the blog post group in the toxicology topic compared to the podcast group (p < 0.01).

Media usage conditions and exit questionnaire responses

The podcast group used computers and mobile devices equally for listening to the media. Podcast users tended to listen to the media in multiple sessions (60%) that were 15-30 minutes in duration on average. Students liked that the podcast “taught us how to approach a clinical presentation and walked us through steps for differential and management”, “was easy to listen to and kept a constant volume level”, and “was good for consolidating information”. In terms of points of improvement for the podcast, a few comments were made regarding the use of acronyms that were not explained fully. For podcast length, 71% of students who used the asthma podcast thought it should be shorter; individual free-text comments suggested that 30 minutes was preferable to most. On the other hand, only 39% of the toxicology podcast users wanted the podcast to be shorter. Multiple comments stated that the longer length was appropriate, as toxicology was a new subject matter that was not reviewed in medical school. The students that did not listen to the podcasts cited lack of time and podcast length to be barriers to media usage.

The blog post group also used computers and mobile devices equally to browse the websites. They tended to read the blog post all at once (72%) rather than in multiple sessions. Students reported taking 25 minutes to read either blog post (average 1,500 words). None of the students thought either blog post should be shorter in length, while a few believed the toxicology blog post could have been longer to provide more background information. This group also stated that toxicology was a new learning topic for them. The blog posts were “like shorter versions of textbooks, and much more directed to students”, “good for copying into my own notes’, and “how I wish my tutorials were laid out”.  Suggestions for improvement included more pictures and diagrams.

**Figure 4:**
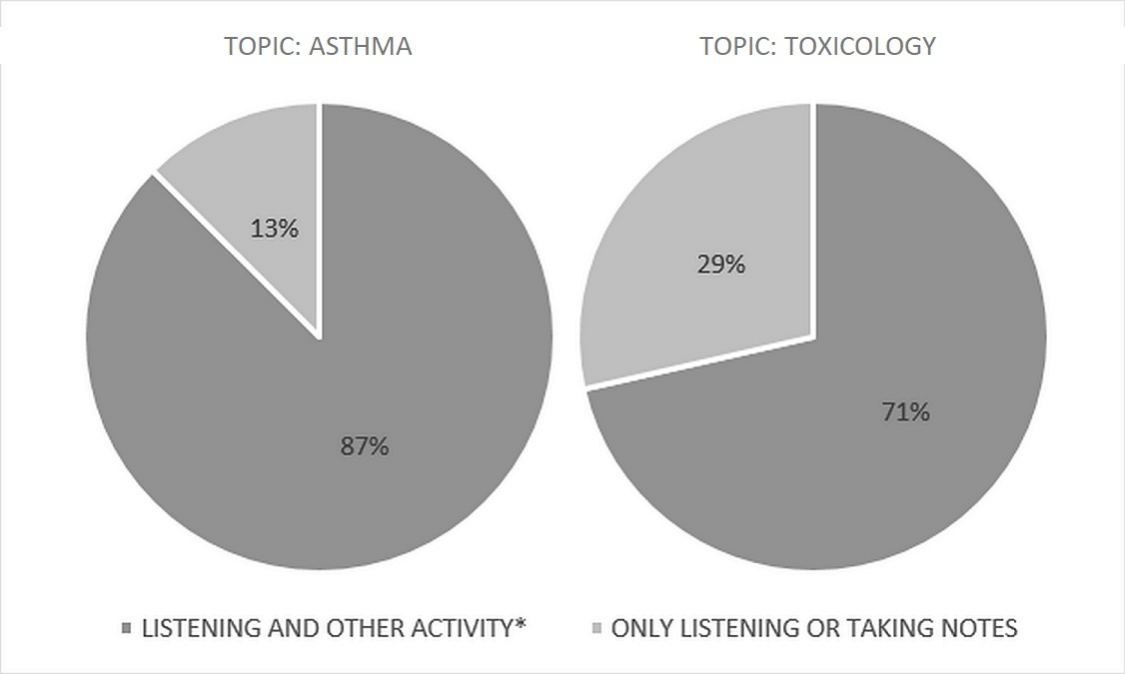
Podcast usage conditions by undergraduate medical students by topic Other activities include driving, physical activities, such as exercising, walking, chores, and eating.

Usage patterns differed between media types. The podcast group tended to engage in multiple activities (i.e., listening as well as another activity) while using the learning materials (79%; n = 22/28) (Figure 4).

**Figure 5:**
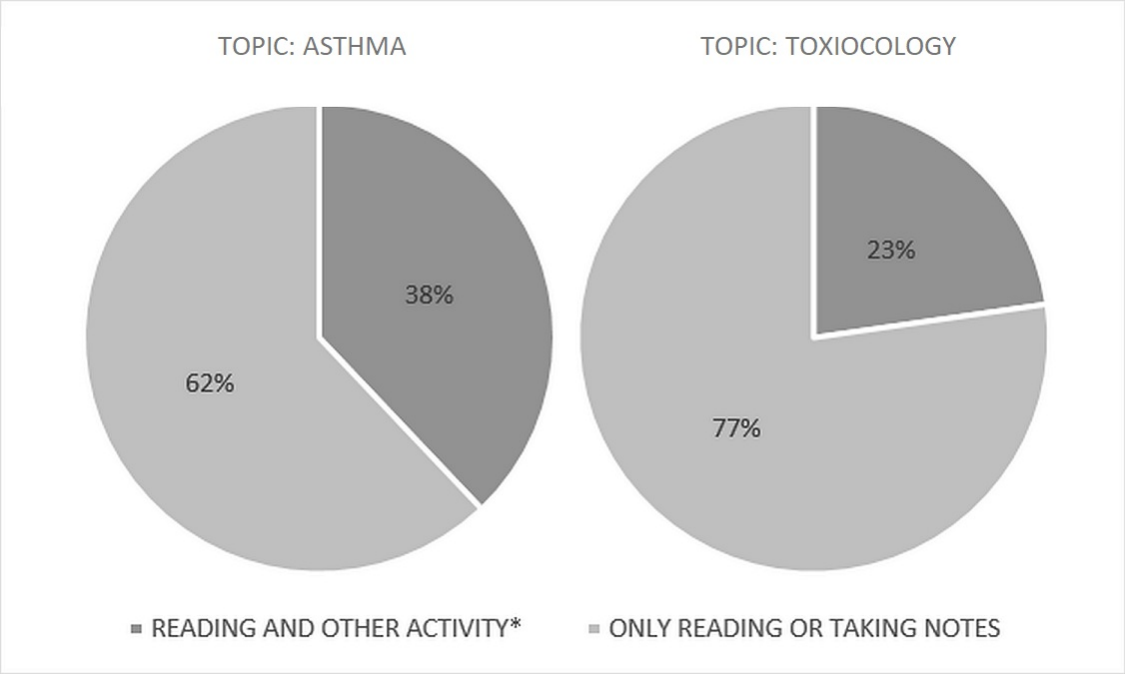
Blog post usage conditions by undergraduate medical students by topic Other activities include physical activities, such as exercising, walking, and eating.

The most common activities were driving, exercising, and eating. Of the two podcasts, students were more likely to only be listening or taking notes on the toxicology topic (29% versus 13%). Blog post users, on the other hand, only participated in multiple activities 31% of the time (n = 9/29) (Figure 5).

These activities included exercising, walking, and eating. The toxicology topic had more students only reading or taking notes compared to the asthma topic (77% versus 62%).

## Discussion

FOAM resources, such as podcasts and blog posts, are increasingly being used by medical learners to supplement their learning [5, 8, 13-15]. It has been suggested that the “Digital Natives” [23] – students born after 1982 – even learn differently compared to previous generations, being more comfortable with audio and visual resources [3, 24]. Advantages of podcasts over traditional didactic lectures include ease of use, ability to learn on-the-go, and the means of reviewing and pausing at the user’s leisure. In terms of blog posts, these are also easily accessible, portable, and easy for students to incorporate into existing notes. While FOAM resources have not yet replaced lectures and textbooks, studies suggest that they may aid in knowledge acquisition and retention in medical learners in combination with traditional resources [10, 18, 20]. Curricular developers in medical schools may consider curating suggested podcasts and blog posts for their students to complement existing didactics.

To the best of our knowledge, this is the first study that compares the two types of media to each other, as well as the first study to evaluate knowledge acquisition with blog posts. A review of the literature produces various publications with undergraduate medical students. These can be separated into studies evaluating podcasts alone for learning [17-18], studies with cross-over to lectures [11, 19], and the study by Back and colleagues comparing podcasts to traditional texts [10]. Of note, these studies generally used a podcast with visual components, e.g., PowerPoint presentations, while the current study used audio-only podcasts.

Both podcasts and blog posts improved knowledge on the two topics in our study. Interestingly, students in both groups were able to achieve a larger gain in knowledge with the toxicology topic as compared to asthma. According to the students, toxicology was a topic that was not covered directly in the undergraduate medical curriculum, whereas asthma was more familiar and covered in respiratory physiology lectures. This suggests that either podcast or blog post could be effective for learning a novel concept rather than merely for review of previously introduced material. While our study showed a similar improvement in test scores for the asthma topic, the blog post group enjoyed a statistically significant larger gain of knowledge for the toxicology topic when comparing students that completed both post-intervention tests. It may be that blog posts are better for learning a novel topic compared to podcasts, but further research is required.

Secondary objectives in the current study were to gain data on the extracurricular conditions in which students use these audio and text media. Literature suggests that multitasking (or more accurately, ‘task switching’) hinders learning [25-26]. In regards to audio-only media specifically, a study by Doolittle and Mariano found that students in a stationary position outperformed students in a mobile environment when using a portable digital media player [27]. However, Coens and colleagues conducted two studies with conflicting results regarding podcast usage and performing a secondary task while learning [28]. In the current study, even though students were more likely to be multitasking when listening to podcasts, their final post-intervention results were comparable to the blog post group. Additionally, while the blog post group was more diligent in taking notes while reviewing the material, this extra step did not appear to have a significant effect on knowledge acquisition. A possible explanation for this observation is that more students were exercising while listening to the podcasts compared to reading the blog posts, which may positively influence cognition as suggested in prior studies in the literature [29-30].

In terms of other considerations for podcast and blog post development, content creators should be conscious of the length of time required to use the resource. A podcast of 30 minutes or less or a blog post of 1,000 words is more acceptable to learners based on our study. Depending on the topic, users may be amenable to longer media when learning about a novel topic. “Approach to” types of topics may be the most popular with undergraduate medical students. Finally, to consolidate learning, multiple choice questions may be generated to promote retention of knowledge, in addition to a summary or handout sheet. These findings were similar to the conclusions of the study by Chin and colleagues [21].

Limitations to our study include using the same questions for both pre- and post-intervention tests. While students may have remembered the initial questions and were therefore primed for answering the exit quiz, we believe that this does not make a significant difference in our primary objective of determining if there was a difference in learning with the two media types, as both groups would experience the same priming effect. The results of this study were also based on a small number of medical students from a single medical school using two topics that are emergency medicine (EM)-based. Future studies should incorporate other disciplines since specialties, such as radiology and pathology, may not benefit from audio-based resources. Finally, as there was no control group in the study (i.e., a separate study arm that would consist of students without access to the learning materials), participants may have learned the topics in the medical school curriculum.

## Conclusions

This study suggests that use of podcasts and blog posts by undergraduate medical students is useful for extracurricular knowledge acquisition. Students preferred podcasts with a duration of 30 minutes and blog posts consisting of 1,000 words or less. They also frequently multitask when listening to podcasts, while opting to write notes or only read when using blog posts. Other activities commonly performed when using these media were driving, exercising, doing chores, and eating. Content creators should consider these usage conditions when generating learning material and should include summary handouts and practice questions when possible.
